# Titin, a Central Mediator for Hypertrophic Signaling, Exercise-Induced Mechanosignaling and Skeletal Muscle Remodeling

**DOI:** 10.3389/fphys.2016.00076

**Published:** 2016-03-01

**Authors:** Martina Krüger, Sebastian Kötter

**Affiliations:** Institute of Cardiovascular Physiology, Heinrich Heine University DüsseldorfDüsseldorf, Germany

**Keywords:** passive tension, connection, striated muscle, posttranslational modification, hypertrophic signaling

## Abstract

Titin is a giant scaffold protein with multiple functions in striated muscle physiology. Due to the elastic I-band domains and the filament-like integration in the half-sarcomere titin is an important factor for sarcomere assembly and serves as an adaptable molecular spring that determines myofilament distensibility. Protein-interactions e.g., with muscle ankyrin repeat proteins or muscle LIM-protein link titin to hypertrophic signaling and via p62 and Muscle Ring Finger proteins to mechanisms that control protein quality control. This review summarizes our current knowledge on titin as a central node for exercise-induced mechanosignaling and remodeling and further highlights the pathophysiological implications.

## Titin—a giant multifunctional spring

**Figure 1:**
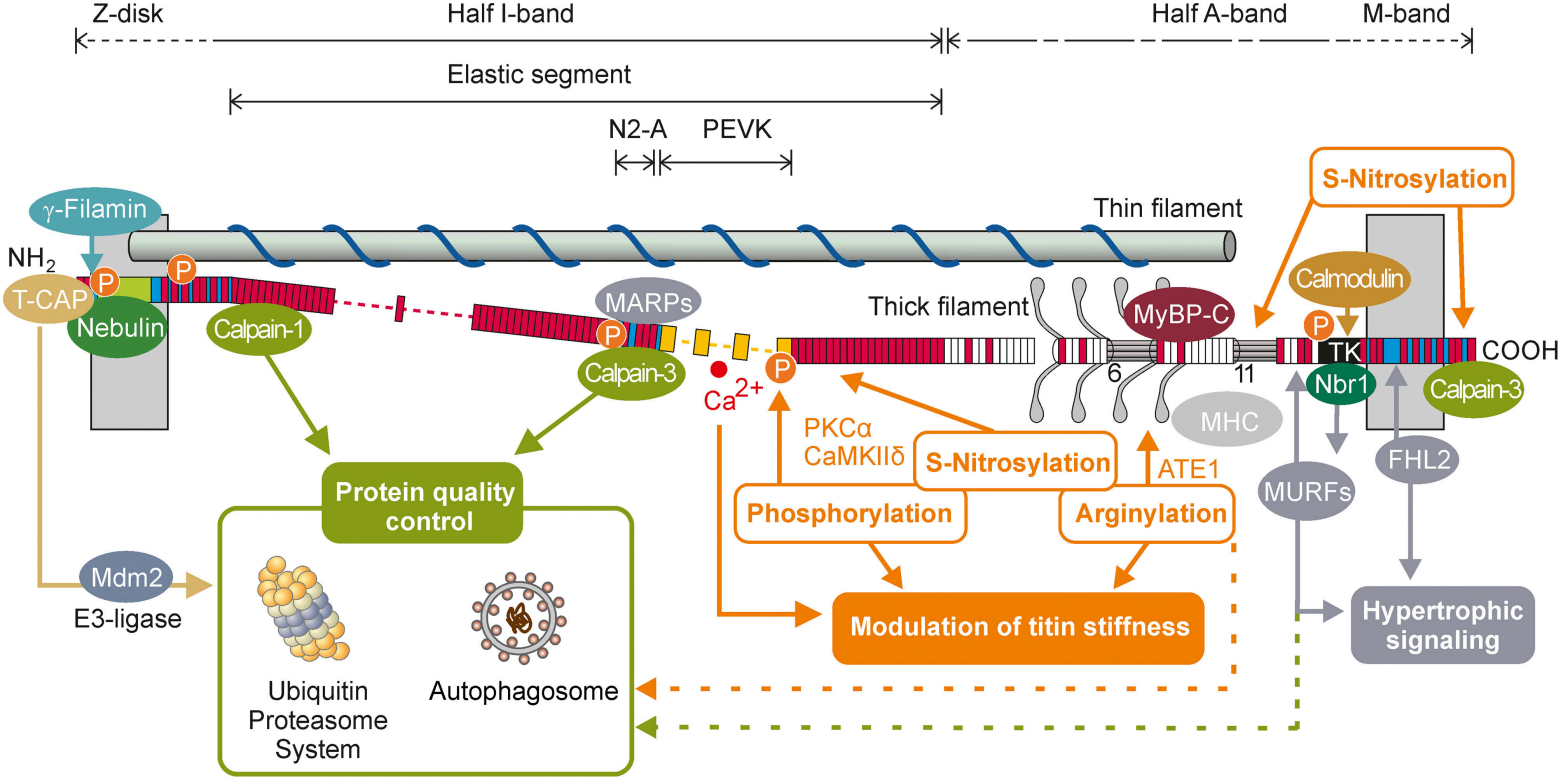
**Scheme of titin-domain architecture (N2A-isoform of human skeletal muscle) in a half-sarcomere with selected titin ligands and titin-based signaling**. Numbers indicate six superrepeats with seven modular domains and 11 superrepeats with 11 modular domains within the thick filament-associated A band titin. T-CAP, telethonin (titin-cap); FHL2, four-and-a-half LIM domain protein 2; Nbr1, neighbor-of-BRCA1-gene-1; MURFs, Muscle RING finger proteins 1/2; Mdm2, mouse double minute-2 protein; MARPs, muscle ankyrin repeat domain proteins; PKCα, Ca2+-dependent protein kinase α; CaMKII δ, Ca2+/calmodulin-dependent protein kinase II δ; MyBP-C, myosin-binding protein C; MHC, myosin heavy chain; ATE1, Arginine-tRNA-protein transferase 1; P, phosphorylation site; Immunoglobulin-like domains (white bars); Fibronectin-like domains (red bars), unique sequences (blue bars), PEVK-elements (yellow bars), Z-repeats (green bar), TK, titin kinase domain.

The backbone of the sarcomere is composed of three filament systems: the myosin-based thick filament, the actin-based thin filament, supplemented with the regulatory protein tropomyosin and the troponin complex, and the titin filament. Titin is a giant protein that spans a half-sarcomere from the Z-disc to the M-line. Differential splicing of the titin gene results in numerous species- and muscle-specific titin isoforms. The skeletal muscles isoform type is called N2A titin (3.3–3.7 MDa) and is expressed as many muscle-specific splice variants (Freiburg et al., 2000; Neagoe et al., 2003; Prado et al., 2005). The titin filament is sequentially arranged by immunoglobulin-like domains (Ig-domains), fibronectin-type-3 domains and several so-called unique sequences (us) (Bang et al., 2001). The NH_2_-terminal end of titin is anchored in the sarcomeric Z-disc via nebulin or the cardiac isoform nebulette (Witt et al., 2006), α-actinin 2 (Labeit et al., 2006), and telethonin (T-CAP) (Granzier and Labeit, 2004; Miller et al., 2004; Lange et al., 2006). In the I-band part skeletal muscle titin is composed of a series of proximal Ig-domains, the N2A-domain (including the N2-A unique sequence), the PEVK domain [high abundance of proline (P), glutamic acid (E), valine (V), and lysine (K)] and the distal Ig-domains. The I-band part is sequentially extended during sarcomere stretch and represents the main elastic segment of titin (Linke et al., 1996, 1999; Trombitas et al., 1998; Li et al., 2002). The largest part of the titin molecule lies within the A-band (Bang et al., 2001) and is tightly associated to myosin and myosin binding protein C (MyBP-C; Tskhovrebova and Trinick, 2004; Lange et al., 2006). The M-band portion of titin contains several inserted sequences and the titin-kinase-domain (Figure 1; Bang et al., 2001; Gautel, 2011). Due to its huge size titin is a scaffolding protein important for sarcomerogenesis and myofibrillar assembly (Ehler and Gautel, 2008; Tskhovrebova and Trinick, 2010). Its central position in the sarcomere and the tight association to myosin are the basis for titin's role in maintaining the structural integrity of the sarcomere during the relaxation-contraction cycle. By reversible extension of the elastic I-band domains upon mechanical stretch titin acts as a molecular spring and defines the passive mechanical properties of the myofilaments (Granzier and Wang, 1993; Bartoo et al., 1997).

As differential splicing of titin mainly affects the I-band portion of the molecule, the size of the expressed titin isoform represents a major determinant of titin-based myofilament stiffness (Prado et al., 2005). More dynamically, titin stiffness is modulated via posttranslational modifications in the elastic I-band region, e.g., by phosphorylation of a so-called *unique sequence* (us) within the N2-B region (N2-B*us*; cardiac muscle specific) and PEVK (cardiac and skeletal muscle). To date, phosphorylation sites have been identified for cAMP-dependent protein kinase (PKA; Yamasaki et al., 2002; Krüger et al., 2009; Kötter et al., 2013), cGMP-dependent protein kinase (PKG; Krüger et al., 2009; Kötter et al., 2013), Ca^2+^-dependent protein kinase C α (PKCα; Hidalgo et al., 2009), extracellular signal regulated kinase 1/2 (Erk1/2; Raskin et al., 2012), and Ca^2+^/calmodulin-dependent protein kinase II delta (CaMKIIδ; Hamdani et al., 2013; Hidalgo et al., 2013). Phosphorylation of the cardiac specific N2-B*us* by PKA, PKG, and CaMKIIδ increases the persistence length of this region and thereby decreases titin-based passive stiffness (Krüger et al., 2009; Hamdani et al., 2013). In contrast, phosphorylation of the PEVK domain by PKCα decreases the persistence length of the PEVK region and causes an increase in titin stiffness (Hidalgo et al., 2009). There is emerging evidence for an important role of oxidative stress in regulating striated muscle elasticity (Beckendorf and Linke, 2015). Oxidate stress induces reversible S-glutathionylation of cryptic cysteines of titin, which has been identified as a potent mechanism to reduce titin stiffness depending on the unfolding status of the immunoglobulin domains (Alegre-Cebollada et al., 2014). It has further been suggested that oxidative stress may induce S-nitrosylation of sarcomeric proteins including titin and thereby depress myofilament Ca^2+^ sensitivity in intact cardiomyocytes (Figueiredo-Freitas et al., 2015). Whether the reported S-nitrosylation affects titin-based passive stiffness remains to be investigated. Just recently it has been recognized that skeletal muscle titin can be arginylated on five sites in the A-band region, where titin associates with myosin, myosin binding protein C (MyBP-C), and myomesin. Genetic depletion of arginyltransferase (ATE1) and loss of titin arginylation resulted in a significant reduction in titin-based passive stiffness (Leite et al., 2016). Future studies will have to establish whether titin-arginylation may serve as a degradation signal for selective autophagy, as recently suggested (Cha-Molstad et al., 2015). However, further analysis of titin modification will doubtlessly result in the identification of many more modifications of titin and their role in striated muscle physiology and pathophysiology in the near future.

## Titin—a mechanosensor for hypertrophic signaling and protein quality control

Considering its gigantic size and the central position within the sarcomere, titin is a very potent and likely candidate to sense alterations of mechanical load. Interactions with more than 20 proteins have been shown until today linking titin to diverse signaling pathways (Figure 1). These interactions have mainly been found in the Z-disc, the elastic I-band and the M-band including the titin kinase (reviewed in Linke and Krüger, 2010; Kötter et al., 2014a). For a broader overview on this topic the following paragraph includes data from the more extensively studied cardiac muscle that may also apply to skeletal muscle.

Located within the Z-disc, a complex formed by muscle LIM protein (MLP), titin Z1Z2 domains and telethonin (T-CAP) has been proposed to act as a mechanical stretch sensor (Knöll et al., 2002, 2011). MLP contains a nuclear translocation signal that allows MLP to shuttle between the cytoplasm and the nucleus. In cardiac myocytes induction of biomechanical stress increased nuclear localization of MLP and was reported to mediate the onset of hypertrophic remodeling processes (Boateng et al., 2009). Activated MLP/T-CAP/titin complex has also directly been linked to hypertrophic signaling by interaction with the calcineurin/NFAT cascade, which results in translocation of NFAT to the nucleus and subsequent activation of target genes e.g., cytokines (Frey et al., 2000; Olson and Williams, 2000). In C2C12 myoblasts MLP has been shown to enhance skeletal myogenesis and to be essential for terminal myocyte differentiation (Kong et al., 1997). Ig-domains of titin's N2A-domain have been shown to interact with the three homologous muscle-ankyrin-repeat proteins (MARPs), cardiac ankyrin repeat protein (CARP), diabetes related ankyrin repeat protein (DARP), and ankyrin-repeat-domain protein-2 (Ankrd2; Miller et al., 2003; Witt et al., 2005b). MARPs are suggested to fulfill important roles in transcriptional regulation, myofibrillar assembly, cardiogenesis and myogenesis, and their altered expression in neuromuscular disorders and cardiovascular diseases further imply a substantial role in pathological processes (reviewed in Kojic et al., 2004). However, the importance of MARPs in regulating the above mentioned processes is still under debate, as recent evidence from knock-out studies demonstrated that all three members of the MARP family are dispensable for normal cardiac function (Bang et al., 2014). Interestingly, in skeletal muscle, knock-out of all three MARPs resulted in more compliant muscle fibers with longer resting sarcomere lengths. Such fibers expressed a longer titin isoform than wild-type animals, indicating that MARPs and their interaction with titin may play a role in the passive mechanical behavior of muscle (Barash et al., 2007). An additional hotspot for titin-mediated hypertrophic signaling is the M-band region of the molecule, especially the titin kinase (TK) domain located in the M-band periphery. In its activated state TK has been shown to directly interact with the ubiquitin-associated zinc-finger protein neighbor of-BRCA1-gene-1 (Nbr1), which forms a signaling complex with p62/SQSTM1 and the muscle ring finger proteins MuRF1, MuRF2, and MuRF3 (Lange et al., 2005). Knock down of both MuRF1 and 2 results in cardiac and skeletal muscle hypertrophy suggesting an inhibitory effect of MuRFs on hypertrophic signaling (Witt et al., 2008).

Via its binding partners titin is not only linked to hypertrophic signaling but also to protein-quality-control and the ubiquitin-proteasome-system. T-CAP interacts with the E3-ligase Mdm2 (Tian et al., 2006). Titin M-band domains A168-170 interact with MuRF-1 and 2, E3 ligases, which have been shown to bind to several muscle proteins including troponin I, troponin T, nebulin, and telethonin, and may thereby mediate their degradation (Pizon et al., 2002; Centner et al., 2003; Gregorio et al., 2005; Witt et al., 2005a). In addition to its role in hypertrophic signaling the Nbr1/p62/SQSTM1 complex targets ubiquitin chains to substrate proteins and thereby promotes their proteasomal degradation (Seibenhener et al., 2007). Via interaction with the autophagosomal membrane anchor LC3, p62, and Nbr1 target polyubiquitinated proteins to the autophagic protein turnover machinery (Pankiv et al., 2007; Waters et al., 2009). This is an important link between the two main degradation systems within the cell.

The spring region of titin has further been reported to associate with protective components of the protein quality control system. A complex formed by titin's N2A-domain, the SET and MYND domain containing protein 2 (Smyd2) and HSP90 was identified to protect titin from degradation (Donlin et al., 2012). In addition, small heat shock proteins (sHSPs) αB-crystallin and HSP27 protect the titin filament from interfilament-aggregation at the N2A-domains (Kötter et al., 2014b).

## The role of titin in exercise-induced remodeling of skeletal muscle

It has long been recognized that eccentric contraction initiates a cascade of events that eventually leads to cytoskeletal and sarcomeric disruption, which is followed by invasion of immune cells as part of the inflammatory response of the damaged muscle (Fridén and Lieber, 1998). An early manifestation of the cytoskeletal damage is a dislocation and loss of the intermediate filament desmin thus disturbing its function as a linker between the myofibrillar Z-disk and the cytoskeletal structures of the cell (Lieber et al., 1994). Sarcomeric injuries include Z-disk streaming, A-band disorganization, and hypercontracted regions significantly impact the mechanical performance of the skeletal muscle fibers (Fridén and Lieber, 1998). Histological stainings of exercised fibers already indicated changes in the intrasarcomeric abundance of titin (Lieber et al., 1996). More recently, a study using immunogold staining demonstrated that a single eccentric exercise bout induces a dislocation of the COOH terminus of titin toward the A band and H zone of the sarcomere, indicating exercise-induced stretch or fragmentation of the titin filament (Macaluso et al., 2014).

Since fiber lesions were still observed several days after the exercise it was hypothesized that some of the changes in the sarcomeric structure represent an intermediate stage of sarcomerogenesis rather than persistent signs of fiber injury (Yu et al., 2003). Interestingly, within the first 2 days after a single eccentric exercise unit titin mRNA expression remains unaltered (Lehti et al., 2009) and the protein contents of nebulin and titin were even significantly reduced (Trappe et al., 2002). This finding possibly indicates an increased turnover of sarcomeric proteins in response to fiber injury. At the same time, eccentric exercise was shown to rapidly elevate the expression of titin-interacting proteins involved in hypertrophic signaling. Among the altered proteins were the members of the MARP family (CARP, DARP, Ankrd2) and MLP (Lehti et al., 2009; Figure 1). Activation of these proteins probably represents an initial step toward adaptative remodeling of the exercised muscle and may also be involved in the initiation of sarcomerogenesis and fiber repair (McKoy et al., 2005; Shi et al., 2005). Whether such activation of titin-interacting proteins is directly mediated by exercise-induced stretching or fragmentation of the titin filament remains to be elucidated. It is also unclear, whether titin degradation is performed by the proteasome or the autophagosomal system, or a combination of both. However, the firm integration of titin in the sarcomeric structure implies some kind of predigestion of the molecule to allow subsequent proteasomal degradation. Such pre-digestion of muscle proteins has previously been demonstrated in septic muscle and was related to the activity of the calpain family of proteases (Williams et al., 1999). It has been demonstrated that acute eccentric exercise altered the expression levels of Calpain-2, and not of the titin-associated Calpains 1 or 3 (Lehti et al., 2009), but it significantly increased the autolysis and subsequent activation of Calpain-3 (Macaluso et al., 2014). Recent advances revealed that calpain-3 activation is not mediated by stretch alone, but is facilitated by the Ca^2+^-binding protein calmodulin and requires elevated levels of resting [Ca^2+^],which occur e.g., during eccentric exercise (Ermolova et al., 2015).

**Figure 2:**
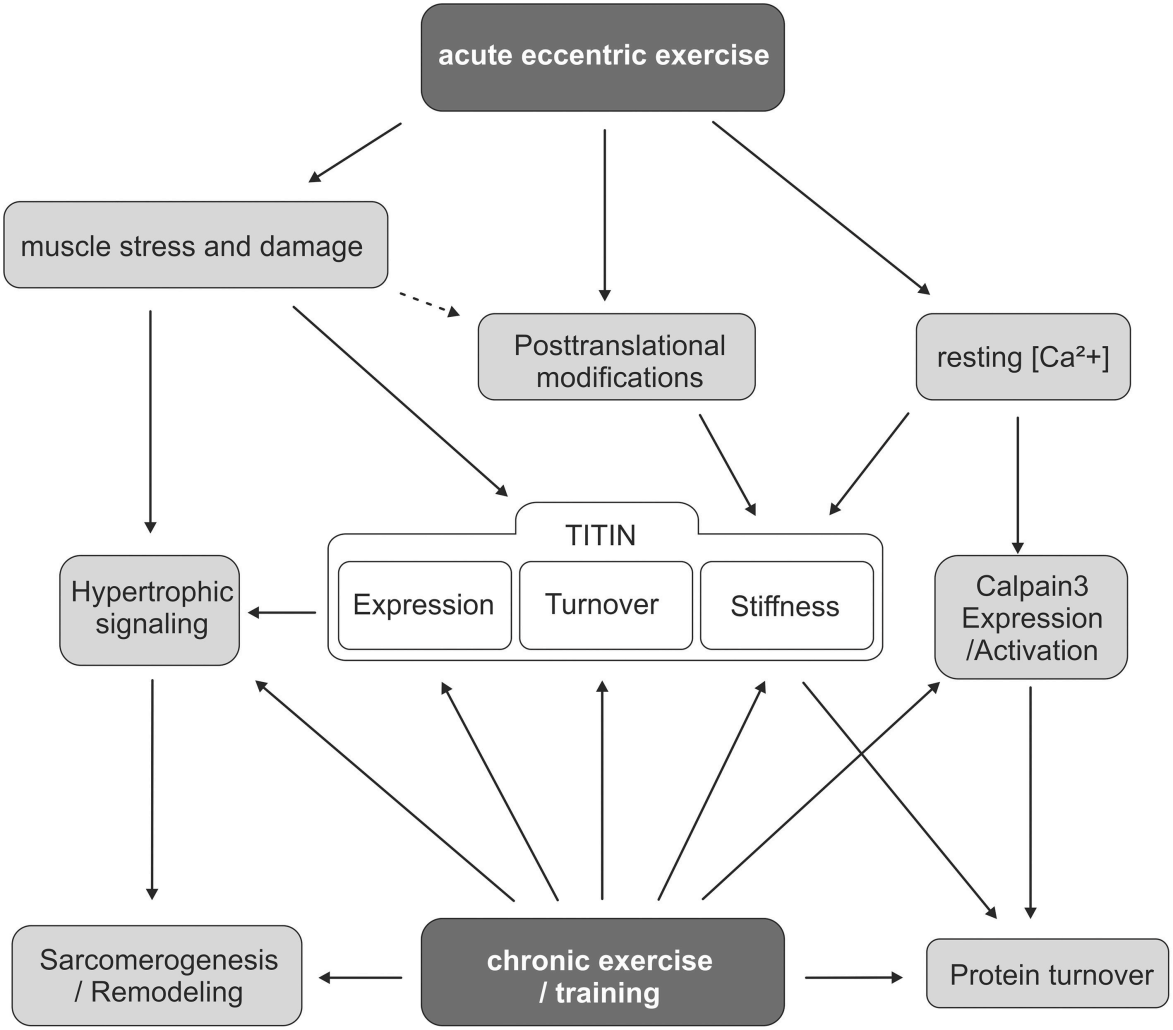
**Scheme of titin involvement in muscle adaptation after acute and chronic exercise**. The scheme summarizes the current knowledge and the different levels of stimuli that modulate titin turnover and titin-based myofilament stiffness in response to acute or repeated exercise. It further highlights the interplay of different signaling pathways that link titin to exercise-induced remodeling processes.

Taken together, the sarcomeric damage caused by acute eccentric exercise initiates calpain-mediated degradation of disrupted sarcomeric filaments, including titin, and at the same time it mediates a possibly titin-related hypertrophic response that supports sarcomerogenesis and fiber repair (Figure 2). This hypertrophic response is further enhanced by repeated exercise, which results in adaptive muscle remodeling and has been associated with increased expression of structural sarcomeric proteins including titin, desmin, and dystrophin (Teran-Garcia et al., 2005; Bellafiore et al., 2007; Lehti et al., 2007).

Repeated exercise has also been suggested to modulate titin turnover. A significant reduction in the titin degradation product T2 was found in the diaphragm of rats that underwent regular running exercise for a period of several weeks (Hidalgo et al., 2014). The appearance of the T2 band is likely due to proteolysis of full-length titin in and near the PEVK region of titin, which results in a large titin (T2) fragment that contains the A-band and distal Ig-segments of titin (Helmes et al., 1996). A previous report showed that in biopsies of vastus lateralis muscle from different athletic populations the relative amount of T2 was much lower than in non-athletes (McBride et al., 2003). However, interpretation of the T2 abundance is not that simple, as a low abundance of the degradation product T2 may be explained by increased sarcomere stability with reduced titin turnover, but it could also be the result of an increased titin turnover rate that leads to immediate processing of the large T2 fragment. Hence, the importance of changes in the abundance of the T2 fragment needs to be further established.

It seems likely that degraded titin does not exert a substantial amount of passive tension on the affected sarcomere. But how does exercise modify titin-based myofilament stiffness in the remaining, non-injured part of the muscle fiber? Recent studies have demonstrated that exercise induces posttranslational modifications of titin that affect titin stiffness and may modulate the mechanical muscle performance. Rats that have been subjected to a single eccentric exercise bout of 15 min. showed a significant increase in the relative phosphorylation of the elastic PEVK-domain of titin, which is expected to result in higher titin-based stiffness of vastus lateralis muscle (Müller et al., 2014). Similar observations have been reported for mice after 3 weeks of voluntary running wheel exercise. Diaphragm samples from these mice also showed increased titin PEVK phosphorylation, thus indicating elevated titin stiffness (Hidalgo et al., 2014; Figure 2). The study further provided an additional explanation for previously reported increases in titin expression after exercise (Bellafiore et al., 2007). It has been calculated that in a normal sarcomere one thick filament (Myosin heavy chain, MHC) can be flanked by a maximum of six titin molecules (Granzier and Irving, 1995). Hidalgo and colleagues demonstrated that under non-exercised conditions the titin: MHC ratio is only ~3:1 and is raised in response to chronic exercise training to ~5:1 (Hidalgo et al., 2014). This too is considered to translate into elevated myofilament stiffness. Calcium associates to the PEVK element of titin and reduces the bending rigidity of this domain (Labeit et al., 2003). As eccentric exercise elevates resting [Ca^2+^], titin-based myofilament stiffness may be further increased upon Ca^2+^-binding. Interestingly, elevated [Ca^2+^] increases titin-based passive stiffness in skeletal but not in cardiac muscle (Cornachione et al., 2016) This exercise-induced increase in titin-based myofilament stiffness may indeed be beneficial for the stressed muscle as it supports titin's role in maintaining the central location of the A-band within the sarcomere. Titin stiffening could therefore improve sarcomere integrity during muscle exercise and thereby promote the effectiveness of accelerated contraction-relaxation cycles.

## Pathophysiological implications

Titin's emerging role in mediating some of the adaptative processes induced by muscular exercise also harbors some important implications for muscle diseases, especially for muscle-disuse and atrophy. In rat gastrocnemius muscle paralyzed with botulinum toxin A (Btx-A) for 3 weeks the proportion of type IIa and IIx MHC were significantly increased while the proportion of type IIb MHC was decreased. At the same time titin content was significantly reduced (Legerlotz et al., 2009). These findings indicate major changes in the active and passive mechanical properties of the paralyzed muscle. In fact, human diaphragm paralyzed for only 2 h during thoracic surgery already presented selective muscle fiber weakness and a reduction in the force-generating capacity (Welvaart et al., 2011). It was further demonstrated that mechanical ventilation activated the ubiquitin-proteasome pathway (Hooijman et al., 2015). A rat model of mechanically ventilated rats confirmed the detrimental effect of even short-term external ventilation on diaphragm performance. In response to mechanical ventilation the tested diaphragms displayed a marked reduction of MHC content and active force generation, as well as a decreased phosphorylation status of titin and significantly reduced passive force generation upon stretch (van Hees et al., 2012). These changes occurred within the first 24 h of mechanical ventilation and could therefore represent an initial step toward muscle atrophy. The results of these studies are interesting also from a therapeutic point of view as altered titin phosphorylation and titin stiffness could possibly be targeted pharmacologically, and may therefore represent a potent tool to improve or even prevent muscle weakness in response to mechanical ventilation.

## Conclusion

The currently available literature demonstrates that titin has multiple roles in exercised skeletal muscle. In response to exercise-induced tissue damage titin itself is subjected to dislocation and fragmentation, and probably mediates important steps toward adaptive hypertrophic signaling. The remaining titin filaments are stiffened via posttranslational modification and may thereby improve sarcomere stability and integrity and contribute to exercise induced force enhancement. The delicate network of protein modifications is still under intensive investigation and the constantly improving proteomic analyses will certainly help to improve our understanding of skeletal muscle function and regulation in physiological and pathophysiological conditions. Further studies are needed to unravel the precise role of titin as a signaling node in exercise-induced hypertrophy and it needs to be tested whether titin could be a potential target of pharmacological or physiological intervention in order to improve muscle function in pathological settings such as disuse-induced muscle atrophy.

## Author contribution

All authors listed, have made substantial, direct and intellectual contribution to the work, and approved it for publication.

## Funding

Part of the work reviewed here was financially supported by grants of the German Research Foundation (DFG) to MK (KR 3409/5-1 and SFB 1116 TPA2) and by a grant of the research commission of the University of Düsseldorf to SK.

### Conflict of interest statement

The authors declare that the research was conducted in the absence of any commercial or financial relationships that could be construed as a potential conflict of interest.
